# Correction to “Importance of antenatal identification of small for gestational age fetuses on perinatal and childhood outcomes: A register‐based cohort study”

**DOI:** 10.1111/aogs.70170

**Published:** 2026-02-17

Hertting E, Herling L, Lindqvist PG, Wiberg‐Itzel E. Importance of antenatal identification of small for gestational age fetuses on perinatal and childhood outcomes: a register‐based cohort study. *Acta Obstet Gynecol Scand* 2024;103(1):42‐50.

In the above article, the proportions of “Severe newborn distress” under “Severe adverse outcome^a^” section of Table 1 and the values of “Severe newborn distress” and “Apgar <4 at 5 minutes” under “Severe adverse outcome^a^” section in Table 2 are incorrect. The corrected values are given below:

**TABLE 1:** Background data and major outcome groups for AGA and SGA, and for newborns identified and not identified as SGA before birth.

*n* = 48 843	AGA, *n* = 41 226	SGA, *n* = 5499	*p*‐Value	Non‐ID SGA, *n* = 3649	ID SGA, *n* = 1850	*p*‐Value
Severe adverse outcome^a^						
Severe adverse outcome	1191 (2.9)	368 (6.7)	<0.001	220 (6.0)	148 (8.0)	0.006
Stillborn	71 (0.2)	80 (1.5)	<0.001	60 (1.6)	20 (1.1)	0.099
Severe newborn distress	727 (1.8)	194 (3.5)	<0.001	105 (2.9)	89 (4.8)	<0.001

*Note*: Large for gestational age (*n* = 2118) not included. Continuous variables are presented as means with interquartile range, categorical variables as numbers and proportions.

Abbreviations: AGA, appropriate for gestational age; BMI, body mass index; GA, gestational age, Emergency Cesarean: Including elective cesarean who started up with contractions before planned surgery, Hypertension: blood pressure >140/90 before or during pregnancy; HELLP, Hemolysis, Elevated Liver enzyme, Low Platelet counts syndrome; ID, identified as SGA before birth; NOT ID, not identified as SGA before birth; PE, preeclampsia; SGA, small for gestational age.

^a^ Major outcome groups.

**TABLE 2:** Severe adverse outcome for newborns identified or not identified as SGA before birth.

	Not identified	Identified	cOR	95% CI	aOR^b^	95% CI
	SGA, *n* = 3649	SGA, *n* = 1850				
	*n* (%)	*n* (%)				
Severe adverse outcome*	**220 (6.0)**	**148 (8.0)**	**0.74**	**0.59–0.92**	**1.19**	**0.93–1.53**
Stillborn	**60 (1.6)**	**20 (1.1)**	**1.53**	**0.92–2.55**	**4.79**	**2.63–8.72**
Severe newborn distress^a^	**105 (2.9)**	**89 (4.8)**	**0.59**	**0.44–0.78**	**0.85**	**0.62–1.17**
Apgar <4 at 5 minutes	8 (0.2)	9 (0.5)				

*Note*: Weight deviation at birth was categorized as ≤−15% to >−22% (ref), −22.1% to >−28%, −28.1% to >−33%, >−33%. Body mass index (kg/m^2^) was categorized as <18.5, 18.5–24.9 (ref), 25–30, >30. Educational level was categorized as ≤9 years, 10–12 years, <12 years (ref).

Abbreviations: aOR, adjusted odds ratio; BPD, Bronchopulmonary dysplasia; cOR, crude odds ratio; CPR, cardiopulmonary resuscitation; HIE, Hypoxic ischemic encephalopathy; IVH, intraventricular hemorrhage.

^a^ At least one of the below.

^b^ Adjusted for age, body mass index, educational level, smoking, nulliparity, diabetes type 1, preeclampsia/hypertension, and weight deviation at birth.

This correction affects only the results for the secondary outcome, “severe newborn distress” and does not alter the overall interpretation of the study. The main conclusion remains unchanged.

Apologies for the error.

